# Predictors of Suspected Developmental Delay Identified Through Screening During the First Year of Life Among Special Newborn Care Unit Graduates in Rural North India

**DOI:** 10.7759/cureus.110530

**Published:** 2026-06-09

**Authors:** Girish K Sharma, Vandana Sharma, Sanjeev Chaudhary

**Affiliations:** 1 Pediatrics, Dr. Radhakrishnan Government Medical College, Hamirpur, IND; 2 Anatomical Sciences, Dr. Radhakrishnan Government Medical College, Hamirpur, IND

**Keywords:** low birth weight, neonatal resuscitation, rural india, special newborn care unit, suspected developmental delay, trivandrum developmental screening chart

## Abstract

Background: Expansion of special newborn care units (SNCUs) in India has improved neonatal survival. However, information regarding post-discharge suspected developmental delay outcomes among SNCU graduates remains limited, particularly in rural settings.

Objective: This study aimed to determine the prevalence of suspected developmental delay among infants ≤12 months of age discharged from an SNCU through screening and to identify associated neonatal risk factors.

Methods: This hospital-based prospective follow-up study included 113 infants discharged from the SNCU of a rural tertiary care hospital in North India. Suspected developmental delay was assessed using the Trivandrum Developmental Screening Chart (TDSC), a validated screening tool. Infants who screened positive were considered to have suspected developmental delay. Maternal and neonatal risk factors were recorded. Associations were analyzed using chi-square/Fisher’s exact test and multivariate logistic regression.

Results: Suspected developmental delay based on TDSC screening was identified in eight of 113 infants (7.1%). On univariate analysis, low birth weight (<2.5 kg), prematurity (<37 weeks), maternal hypothyroidism, and neonatal resuscitation were significantly associated with suspected developmental delay (p<0.05). On multivariate analysis, neonatal resuscitation (adjusted odds ratio (OR) 4.12; 95% confidence interval (CI) 1.18-14.32; p=0.026) and low birth weight (adjusted OR 3.21; 95% CI 1.05-9.81; p=0.041) remained independent predictors.

Conclusion: Approximately one in 14 infants discharged from the SNCU showed suspected developmental delay based on screening at one year of age. Neonatal resuscitation and low birth weight were independent predictors. These findings highlight the need for structured developmental screening and follow-up of high-risk neonates within primary care and rural health systems.

## Introduction

India has expanded special newborn care units (SNCUs) under Facility-Based Newborn Care (FBNC) initiatives to improve neonatal survival and reduce neonatal mortality [1,2]. With declining neonatal mortality, there is increasing attention now being directed toward long-term developmental outcomes among high-risk neonatal survivors [2,3]. 

Studies from India have reported a considerable burden of developmental delay among SNCU graduates and other high-risk neonates, particularly among preterm and low birth weight infants [3-5].

Despite improvements in neonatal care, data on post-discharge developmental outcomes among SNCU graduates in India remain limited, particularly in rural settings where structured follow-up services are inconsistent. Most available Indian follow-up data are derived from tertiary care centers, while evidence from rural populations remains scarce [6,7].

Early identification of developmental delay enables timely intervention and may improve long-term cognitive and motor outcomes [8]. Developmental surveillance integrated within primary health-care systems is essential for early detection and management of high-risk infants [9]. National initiatives such as the Government of India’s FBNC program and the Rashtriya Bal Swasthya Karyakram (RBSK) have emphasized screening and early intervention for developmental disorders [1,7]. However, systematic follow-up and developmental screening of infants discharged from SNCUs remain inconsistent in many rural areas [6-8].

Factors such as poor accessibility, limited rehabilitation and early intervention services, inadequate caregiver awareness, and high loss to follow-up rates pose additional challenges to effective developmental surveillance in rural settings.

The present study aimed to determine the prevalence of suspected developmental delay (≤12 months) among infants discharged from an SNCU in rural North India and to identify neonatal predictors associated with adverse developmental outcomes.

## Materials and methods

Study design and setting

This was a prospective hospital-based follow-up study conducted in the Department of Pediatrics at Dr. Radhakrishnan Government Medical College and Hospital, a rural tertiary care teaching hospital in Hamirpur, Himachal Pradesh, India. The study was carried out from July 2025 to January 2026.

Participants

**Figure 1:**
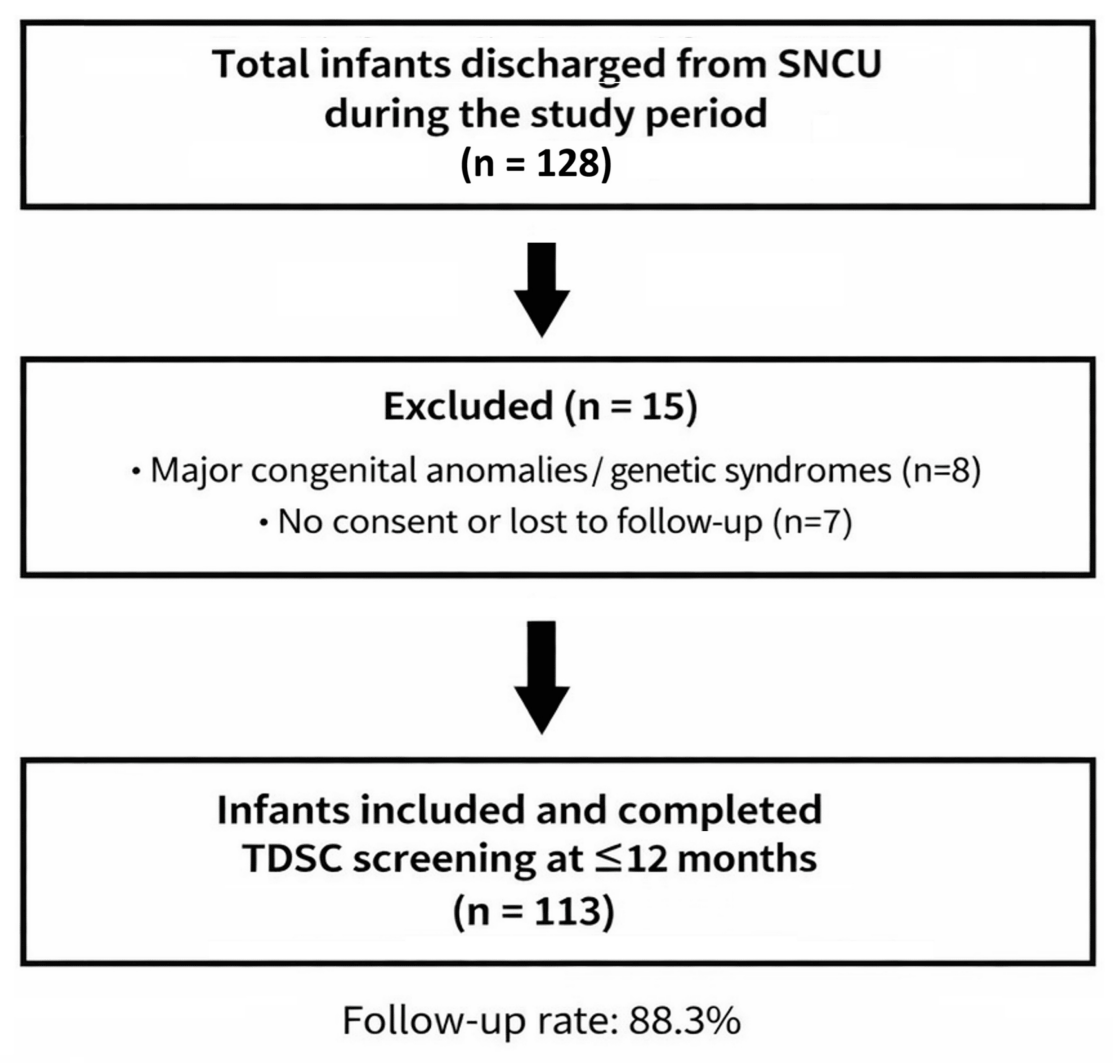
Flow diagram of study participants (STROBE-compliant) A total of 128 infants were discharged from the SNCU during the study period. After applying exclusion criteria, 113 infants were included in the final analysis (follow-up rate 88.3%). SNCU: special newborn care unit; TDSC: Trivandrum Developmental Screening Chart; STROBE: Strengthening the Reporting of Observational Studies in Epidemiology

All infants discharged from the SNCU during the study period were eligible for inclusion. A total of 128 infants were discharged from the SNCU. After excluding eight infants with major congenital anomalies or genetic syndromes and seven infants whose parents did not consent or could not be contacted for follow-up, 113 infants were included in the final analysis (follow-up rate: 88.3%). A flow diagram of study participants is shown in Figure 1.

Sample size

The sample size was determined by the number of eligible SNCU graduates during the study period rather than by a formal a priori calculation. This prospective observational study included all consecutive infants discharged from the SNCU between July 2025 and January 2026 who met the inclusion criteria. Of 128 eligible infants, 113 were included in the final analysis (follow-up rate: 88.3%).

Inclusion and exclusion criteria

Infants discharged from the SNCU were eligible for inclusion if they were ≤12 months of chronological age at the time of assessment and if written informed consent was obtained from the parents or guardians. Corrected age was used for preterm infants during developmental assessment. Infants were excluded if they had major congenital anomalies, known genetic or metabolic syndromes, or if their parents were unwilling or unable to participate in the follow-up assessment.

Data collection

Maternal variables included age, hypothyroidism, hypertension, and anemia. Neonatal variables included birth weight, gestational age, mode of delivery, requirement for resuscitation at birth, and presence of neonatal jaundice.

Low birth weight was defined as birth weight <2.5 kg. Prematurity was defined as gestational age <37 completed weeks. Corrected age was used for preterm infants during developmental assessment. 

Developmental assessment

Developmental screening was performed at ≤12 months of age (mean age at assessment: 7.36 ± 4.41 months) using the Trivandrum Developmental Screening Chart (TDSC), a validated and widely used screening tool in Indian settings [10]. TDSC is a freely available/open-access screening tool. Infants who screened positive on the TDSC were categorized as having suspected developmental delay. All assessments were conducted by a trained pediatrician.

Definition of neonatal resuscitation

Neonatal resuscitation was defined as the requirement for positive pressure ventilation (bag-and-mask positive pressure ventilation or endotracheal intubation) at birth in accordance with Neonatal Resuscitation Program (NRP) guidelines [11]. Infants requiring only tactile stimulation were not classified as having received neonatal resuscitation.

Statistical analysis

Data were analyzed using IBM SPSS Statistics version 26.0 (IBM Corp., Armonk, NY, USA). Continuous variables were expressed as mean ± standard deviation (SD) and categorical variables as frequencies and percentages.

Associations between risk factors and suspected developmental delay were assessed using the chi-square test or Fisher’s exact test, as appropriate. Because of the small number of outcome events (n=8), multivariate logistic regression analysis was restricted to two biologically plausible and statistically significant predictors (low birth weight and neonatal resuscitation) to avoid model overfitting. Adjusted odds ratios (ORs) with 95% confidence intervals (CIs) were calculated. A two-sided p-value <0.05 was considered statistically significant.

The study is reported in accordance with the Strengthening the Reporting of Observational Studies in Epidemiology (STROBE) guidelines for observational studies [12].

Ethical considerations

The study protocol was approved by the Institutional Ethics Committee (Approval No: HFW-H-Dr.RKGMC/Ethics/2025/1 dated 01/07/2025). Written informed consent was obtained from the parents or legal guardians of all participating infants.

## Results

Baseline characteristics

The baseline characteristics of the study participants are presented in Table 1. Of the 113 infants included in the study, 68 (60.2%) were male children and 106 (93.8%) belonged to rural areas. The mean birth weight was 2.73 ± 0.54 kg, with 32 infants (28.3%) classified as low birth weight (<2.5 kg). Twenty-nine infants (25.7%) were born preterm (<37 weeks). Neonatal resuscitation at birth was required in 15 infants (13.3%). Maternal and neonatal risk factors are summarized in Table 2.

**Table 1:** Baseline characteristics of study participants (n=113) Values are presented as numbers (percentages) or mean ± standard deviation (SD). Socioeconomic status was assessed using the modified Kuppuswamy scale [13].

Variable	Value
Male sex, n (%)	68 (60.2%)
Rural residence, n (%)	106 (93.8%)
Lower-middle socioeconomic status, n (%)	97 (85.8%)
Mean age at assessment months, mean ± SD	7.36 ± 4.41
Birth weight (kg), mean ± SD	2.73 ± 0.54
Low birth weight (<2.5 kg), n (%)	32 (28.3%)
Gestational age (weeks), mean ±SD	36.4 ± 2.8
Preterm (<37 weeks), n (%)	29 (25.7%)

**Table 2:** Maternal and neonatal risk factors (n=113) Values are presented as numbers (percentages). Neonatal resuscitation was defined as the requirement for positive pressure ventilation at birth per NRP guidelines [11]. NRP: Neonatal Resuscitation Program

Risk factor	Value
Maternal hypothyroidism, n (%)	17 (15.0%)
Hypertension, n (%)	6 (5.3%)
Neonatal resuscitation, n (%)	15 (13.3%)
Neonatal jaundice, n (%)	83 (73.5%)
Low birth weight (<2.5 kg), n (%)	32 (28.3%)

Prevalence of suspected developmental delay

The developmental outcomes are shown in Table 3. Suspected developmental delay was identified in eight of 113 infants (7.1%) using TDSC screening.

**Table 3:** Developmental outcomes identified through TDSC screening (n=113) Values are presented as numbers (percentages). TDSC: Trivandrum Developmental Screening Chart

Outcome	Value
Suspected developmental delay, n (%)	8 (7.1%)
Normal development, n (%)	105 (92.9%)

Univariate analysis

The association between risk factors and suspected developmental delay on univariate analysis is presented in Table 4. Low birth weight (p=0.041), prematurity (p=0.048), maternal hypothyroidism (p=0.032), and neonatal resuscitation (p=0.021) were significantly associated with suspected developmental delay.

**Table 4:** Association between risk factors and suspected developmental delay on univariate analysis (n=113) Values are presented as numbers (percentages). Associations were analyzed using Fisher’s exact test due to small cell sizes. Crude odds ratios (ORs) with 95% confidence intervals (CIs) are provided from univariate analysis.

Variable	Delay, n (%)	No delay, n (%)	Crude OR (95% CI)	p-value
Low birth weight	5 (62.5%)	27 (25.7%)	4.81 (1.05-22.0)	0.041
Preterm birth	4 (50.0%)	25 (23.8%)	3.20 (0.76-13.4)	0.048
Maternal hypothyroidism	3 (37.5%)	14 (13.3%)	3.90 (0.84-18.0)	0.032
Neonatal resuscitation	3 (37.5%)	12 (11.4%)	4.65 (0.98-22.1)	0.021

Multivariate analysis

**Figure 2:**
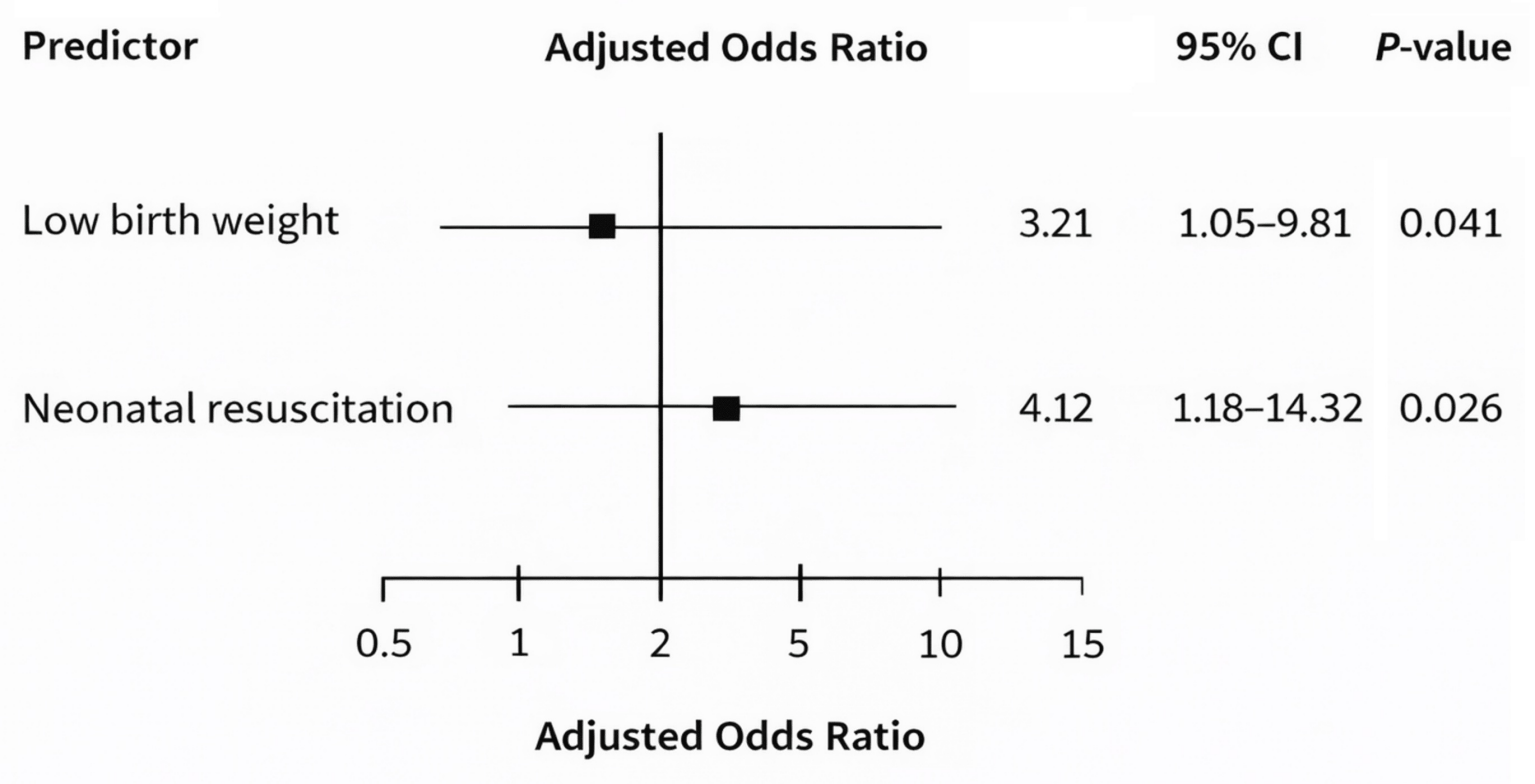
Forest plot showing results of multivariate logistic regression analysis Adjusted odds ratios (ORs) and 95% confidence intervals (CIs) are shown for low birth weight and neonatal resuscitation as independent predictors of suspected developmental delay.

The results of multivariate logistic regression are shown in Table 5 and Figure 2. After adjustment, neonatal resuscitation (adjusted OR 4.12; 95% CI 1.18-14.32; p = 0.026) and low birth weight (adjusted OR 3.21; 95% CI 1.05-9.81; p = 0.041) remained independent predictors of suspected developmental delay.

**Table 5:** Multivariate logistic regression analysis of predictors of suspected developmental delay Adjusted odds ratios (ORs), 95% confidence intervals (CIs), and p-values are shown. The model included only low birth weight and neonatal resuscitation to avoid overfitting, given the small number of outcome events (n=8).

Predictor	Adjusted OR	95% CI	p-value
Low birth weight	3.21	1.05-9.81	0.041
Neonatal resuscitation	4.12	1.18-14.32	0.026

## Discussion

In this prospective hospital-based follow-up study conducted at a rural tertiary care center in North India, suspected developmental delay identified through TDSC screening was observed in 7.1% of infants discharged from the SNCU at ≤12 months of age. These findings highlight the continued vulnerability of high-risk neonates even after surviving the neonatal period [7,8].

The prevalence observed in the present study is lower than that reported in several previous hospital-based Indian follow-up studies on high-risk neonates [7,8]. This difference may be attributed to the use of a screening tool (TDSC), relatively better baseline care in the SNCU, or possible selection bias toward infants who returned for follow-up.

Low birth weight emerged as an independent predictor of suspected developmental delay. This is consistent with existing literature showing that low birth weight and prematurity can adversely affect brain maturation through disturbances in cortical development, myelination, and neuronal connectivity [4,5]. The threefold increased risk observed in this study reinforces the need for targeted follow-up of low birth weight infants after SNCU discharge.

Neonatal resuscitation at birth was the strongest independent predictor, with a fourfold increased risk. Infants requiring resuscitation are more likely to have experienced perinatal hypoxia or other acute insults that can impair early brain development [5]. These infants represent a high-risk subgroup that warrants closer developmental monitoring.

Although prematurity and maternal hypothyroidism were significantly associated on univariate analysis, they did not remain independent predictors in the multivariate model. This is likely due to the small number of outcome events (n=8), which limited statistical power. 

The variable age at assessment within the first year and the use of single-timepoint developmental screening may also have influenced outcome estimation. In addition, some infants with subtle or evolving developmental deficits may have been missed because longitudinal developmental follow-up was not performed.

TDSC used in this study is a practical and validated screening tool suitable for resource-limited settings [10]. It can be administered by trained pediatricians or community health workers, making it valuable for widespread use in rural primary care.

The present findings emphasize the importance of strengthening structured post-discharge follow-up systems for SNCU graduates. Integrating simple developmental screening into routine child health visits under programs such as RBSK can facilitate early identification and timely intervention [8,9]. Family physicians and primary care providers play a pivotal role in this process, as they are often the first point of contact for families in rural areas [2].

Limitations

This study has several limitations. It was a single-center study with a moderate sample size and only eight outcome events, which reduced statistical power and limited the number of variables that could be included in multivariate analysis. The use of TDSC, a screening tool rather than a comprehensive diagnostic instrument (such as the Developmental Assessment Scale for Indian Infants (DASII) or Bayley scale), means the results indicate “suspected developmental delay” rather than confirmed neurodevelopmental impairment [14,15]. Information on important neonatal morbidities (e.g., hypoxic-ischemic encephalopathy (HIE) staging, culture-positive sepsis, duration of ventilation/continuous positive airway pressure (CPAP), and antenatal Doppler findings) was not uniformly available. These limitations should be considered while interpreting the findings. Larger multicenter studies using formal developmental assessment tools are recommended.

## Conclusions

Approximately one in 14 infants discharged from the SNCU in rural North India showed suspected developmental delay based on TDSC screening within the first year of life. Neonatal resuscitation and low birth weight were independent predictors. These findings highlight the need to incorporate structured developmental screening and follow-up into routine care for SNCU graduates, particularly in resource-limited rural settings.
